# Contribution of serum FGF21 level to the identification of left ventricular systolic dysfunction and cardiac death

**DOI:** 10.1186/s12933-017-0588-5

**Published:** 2017-08-18

**Authors:** Yun Shen, Xueli Zhang, Xiaoping Pan, Yiting Xu, Qin Xiong, Zhigang Lu, Xiaojing Ma, Yuqian Bao, Weiping Jia

**Affiliations:** 1 Department of Endocrinology and Metabolism, Shanghai Jiao Tong University Affiliated Sixth People’s Hospital, Shanghai Clinical Center for Diabetes, Shanghai Key Clinical Center for Metabolic Disease, Shanghai Key Laboratory of Diabetes Mellitus, Shanghai Diabetes Institute, Shanghai, China; 2 Department of Cardiology, Shanghai Jiao Tong University Affiliated Sixth People’s Hospital, Shanghai, China

**Keywords:** Fibroblast growth factor 21, Left ventricular systolic dysfunction, Cardiac death

## Abstract

**Aim:**

The relationship between fibroblast growth factor 21 (FGF21) and cardiovascular disease has been well established in recent studies. This study aimed to investigate the relationship between FGF21 and left ventricular systolic dysfunction and cardiac death.

**Methods:**

Two-dimensional echocardiography was used to measure the left ventricular ejection fraction (LVEF) to estimate left ventricular systolic function. The optimal cutoff of FGF21 for identifying left ventricular systolic dysfunction at baseline was analyzed via receiver operating characteristic (ROC) curves. The identification of different serum levels of FGF21 and their association with cardiac death was analyzed via Kaplan–Meier survival curves. Serum FGF21 level was measured by an enzyme-linked immunosorbent assay kit, and serum N-terminal pro-brain natriuretic peptide (NT-pro-BNP) level was determined by a chemiluminescent immunoassay.

**Results:**

A total of 253 patients were recruited for this study at baseline. Patients were excluded if they lacked echocardiography or laboratory measurement data, and there were 218 patients enrolled in the final analysis. The average age was 66.32 ± 10.10 years. The optimal cutoff values of FGF21 and NT-pro-BNP for identifying left ventricular systolic dysfunction at baseline were 321.5 pg/mL and 131.3 ng/L, respectively, determined separately via ROC analysis. The areas under the curves were non-significant among FGF21, NT-pro-BNP and FGF21 + NT-pro-BNP as determined by pairwise comparisons. Both a higher serum level of FGF21 and a higher serum level of NT-pro-BNP were independent risk factors for left ventricular systolic dysfunction at baseline (odd ratio (OR) 3.138 [1.037–9.500], *P* = 0.043, OR 9.207 [2.036–41.643], *P* = 0.004, separately). Further Kaplan–Meier survival analysis indicated an association between both a higher serum level of FGF21 and a higher serum level of NT-pro-BNP with cardiac death in 5 years [RR 5.000 (1.326–18.861), *P* = 0.026; RR 9.643 (2.596–35.825), *P* = 0.009, respectively].

**Conclusions:**

Serum FGF21 level was significantly correlated with left ventricular systolic dysfunction at baseline. Patients with higher serum levels of FGF21 tended to suffer greater risks of cardiac death than patients with lower serum levels of FGF21. The identification of FGF21 and its relationship with left ventricular systolic function and cardiac death were non-inferior to NT-pro-BNP.

## Introduction

Cardiovascular disease comprises all cardiac and vascular disorders, including coronary arterial disease and peripheral vascular disease, among which coronary atherosclerotic heart disease (CAD) is responsible for a large portion [1]. With improvement in the quality of life in developing countries, the incidence of CAD is increasing rapidly. In particular, left ventricular systolic function has gained attention as it can serve as a good indicator for the prognosis of patients with CAD [2]. The left ventricular ejection fraction as measured by two-dimension echocardiography is commonly used to reflect left ventricular systolic function. The first step in the prevention and treatment of CAD is finding an optimal indicator for the early identification of relevant risk factors. The second step is to develop novel therapies for the complications associated with chronic cardiovascular disease including ischemic vascular disease and heart failure. The first step deserves more attention [3].

In a previous cross-sectional study [4], we found that fibroblast growth factor 21 (FGF21), an important cytokine involved in glycol-lipid metabolism in vivo, was closely correlated with CAD as diagnosed by coronary angiography. FGF21 can also be an independent risk factor for CAD. Therefore, this study aimed to investigate the association of FGF21 in left ventricular systolic dysfunction and cardiac death.

## Methods

### Subjects

The study subjects, as reported previously, comprised 253 patients at baseline [4]. We reviewed the subjects’ electronic medical record and found no patients with severe valvular stenosis or insufficiency. Patients were excluded if they lacked echocardiography (n = 31) or laboratory measurement (n = 4) data, and 218 patients were enrolled in the final analysis. Information and outcomes were collected via the electronic medical records system of Shanghai Jiao Tong University Affiliated Sixth People’s Hospital or by telephone calls. The primary outcome was CVD mortality. At baseline, all of the subjects had provided informed consent before coronary angiography. The study protocol has been approved by the Ethics Committee of Shanghai Jiao Tong University Affiliated Sixth People’s Hospital. The median follow-up time was 5.0 years.

### Echocardiography

Echocardiography was performed by a trained specialist with a Philips IE33 ultrasonic device. The left ventricular end-diastolic diameter (LVDd), the interventricular septum dimension (IVST) and the left ventricular posterior wall thickness (PWT) were measured in the parasternal long axis view at the end of the diastolic period. The left atrial diameter (LAD) and the left ventricular end-systolic diameter (LVSd) were measured in the parasternal long axis view at the end of the systolic period. The left ventricular mass (LV mass) was calculated by a formula from Devereux et al. [5] as LV mass = 0.8 × 1.04 × [(LVDd + IVST + PWT)^3^ − LVDd^3^] + 0.6. The LV mass index was calculated as the LV mass divided by the body surface area. The LVEF was measured via Simpson’s method in the apical 4-chamber view. The left ventricular shortening fraction was calculated by (LVDd–LVSd)/LVDd.

### Diagnostic criteria

Graded left ventricular dysfunction was defined according to the recommendations for cardiac chamber quantification by echocardiography in adults published by the American Society of Echocardiography and the European Association of Cardiovascular Imaging as in the normal range (52–72% for men, 54–74% for women), in the mildly abnormal range (41–51% for men, 41–53% for women), in the moderately abnormal range (30–40%) and in the severely abnormal range (<30%) [2].

Diabetes was defined according to the 2010 American Diabetes Association standards as fasting plasma glucose (FPG) of at least 7.0 mmol/L and/or a 2-h postprandial plasma glucose (2-h PG) measurement of at least 11.1 mmol/L and/or a hemoglobin A1c (HbA1c) of at least 6.5% [6].

Dyslipidemia was defined according to the 2016 Joint Committee for Developing Chinese Guidelines on Prevention and Treatment of Dyslipidemia in Adults (JCDCG), including a serum total cholesterol (TC) level ≥5.18 mmol/L; a serum triglycerides (TG) level ≥1.7 mmol/L; a serum low-density lipoprotein cholesterol (LDL-c) level ≥3.37 mmol/L; a serum high-density lipoprotein cholesterol (HDL-c) level <1.04 mmol/L; or having undergone a specific treatment for dyslipidemia [7].

Hypertension was defined according to the World Health Organization (WHO)/International Society of Hypertension (ISH) 2003 criteria as systolic blood pressure ≥140 mmHg or diastolic blood pressure ≥90 mmHg or having undergone a specific treatment for hypertension [8].

Smoking was defined as having at least one cigarette per day and a continuous smoking duration of more than half a year [9].

A family history of CAD is defined as having first-degree relatives who experienced a myocardial infarction, heart failure, stroke, or death due to cardiovascular disease, which are all determined through patients’ self-reports [10].

### Anthropometric and laboratory measurements

The methods used to evaluate body mass index (BMI), waist circumference, blood pressure, fasting and 2-h PG, HbA1c, serum insulin, lipid profile (TG, TC, LDL-c, HDL-c) and C-reactive protein (CRP) detection have been described previously [4]. Serum FGF21 levels were determined using an enzyme-linked immunosorbent assay kit (Antibody and Immunoassay Services, University of Hong Kong) with inter-assay and intra-assay coefficients of variation of 7.8 and 9.1%, respectively. Serum N-terminal pro-brain natriuretic peptide (NT-pro-BNP) levels were determined by a chemiluminescent immunoassay (Cobas 6000, Roche Diagnostics GmbH, Mannheim, Germany) with inter-assay and intra-assay coefficients of variation of 4.6 and 4.2%.

### Statistical analysis

All data were analyzed using SPSS 19.0. Normally distributed data are expressed as the mean ± standard deviation, and skewed data are expressed as medians (inter-quartile range). Serum cutoff values and their corresponding sensitivity and specificity for FGF21, NT-pro-BNP and FGF21 + NT-pro-BNP in the identification of left ventricular systolic dysfunction at baseline were analyzed by receiver operating characteristic (ROC) curve analysis. The areas under the ROC curves (AUC) were compared by pairwise comparison analysis. Multiple logistic regression analysis was conducted to analyze the independent risk factors of left ventricular systolic dysfunction at baseline. Kaplan–Meier analysis was used to plot survival curves, and the event-free survival rates for patients with different FGF21 and NT-pro-BNP levels were compared using the log-rank test. Two-tailed P < 0.05 was defined as statistical significance.

## Results

The characteristics of the study patients at baseline are seen in Table 1. The average age was 66.32 ± 10.10 years old. The average BMI was 24.59 ± 3.46 kg/m^2^. Among the patients, 34.4% (75 individuals) were women who were all postmenopausal. At baseline, there were 45.4% smokers, 40.4% of the patients with diabetes, 76.6% with hypertension and 89.9% with dyslipidemia. There were 42.7% of the patients with family histories of cardiovascular diseases.

**Table 1:** Baseline characteristics

	Patients enrolled (n = 218)
Anthropometric variables	
Sex, male/female	143/75
Age, years	66.32 ± 10.10
Body mass index, kg/m^2^	24.59 ± 3.46
Waist circumference, cm	90.19 ± 10.04
Laboratory variables	
Systolic blood pressure, mmHg	130.0 (120.0–150.0)
Diastolic blood pressure, mmHg	80.0 (70.0–84.2)
Fasting plasma glucose, mmol/L	5.44 (5.00–6.20)
2-h postprandial glucose, mmol/L	8.58 (6.56–11.50)
HbA1c, %	6.2 (5.7–6.7)
HOMA-IR	4.00 (2.89–6.06)
Total cholesterol, mmol/L	4.42 ± 1.11
Triglyceride, mmol/L	1.51 (1.07–2.15)
HDL-c, mmol/L	1.11 ± 0.30
LDL-c, mmol/L	3.01 ± 0.97
C-reactive protein, mg/L	1.32 (0.57–3.82)
FGF21, pg/mL	300.8 (199.1–407.0)
NT-pro-BNP, ng/L	117.9 (52.6–309.2)
Ultrasonographic variables	
LA dimension, mm	37.9 ± 5.5
LV mass index, g/m^2^	85.5 (74.6–98.1)
LVEF, %	62.0 (59.0–66.0)
LVDd, mm	46.0 (44.0–49.0)
LVSd, mm	30.8 (28.6–32.9)
FS, %	33.0 (31.0–36.0)
IVST, mm	10.0 (9.0–11.0)
PWT, mm	9.0 (8.0–9.0)
History	
Smoking, n (%)	99 (45.4)
Diabetes, n (%)	88 (40.4)
Hypertension, n (%)	167 (76.6)
Dyslipidemia, n (%)	196 (89.9)
History of CVD, n (%)	93 (42.7)

Data were expressed as mean ± SD or median (interquartile range). Statistics for categorical variables are number of patients (percentage of patients)

*CVD* cardiovascular diseases, *FGF* fibroblast growth factor, *FS* shortening fraction, *HDL*-*c* high-density lipoprotein cholesterol, *HOMA*-*IR* homeostasis model assessment-insulin resistance, *HbA1c* hemoglobin A1c, *IVST* interventricular septum thickness, *LA* left atrium, *LDL*-*c* low-density lipoprotein cholesterol, *LV* left ventricular, *LVEF* left ventricular ejection fraction, *LVSd* left ventricular end-systolic diameter, *LVDd* left ventricular end-diastolic diameter, *NT*-*pro*-*BNP* N-terminal pro-brain natriuretic peptide, *PWT* posterior wall thickness

### The identification of left ventricular dysfunction at baseline

**Fig. 1:**
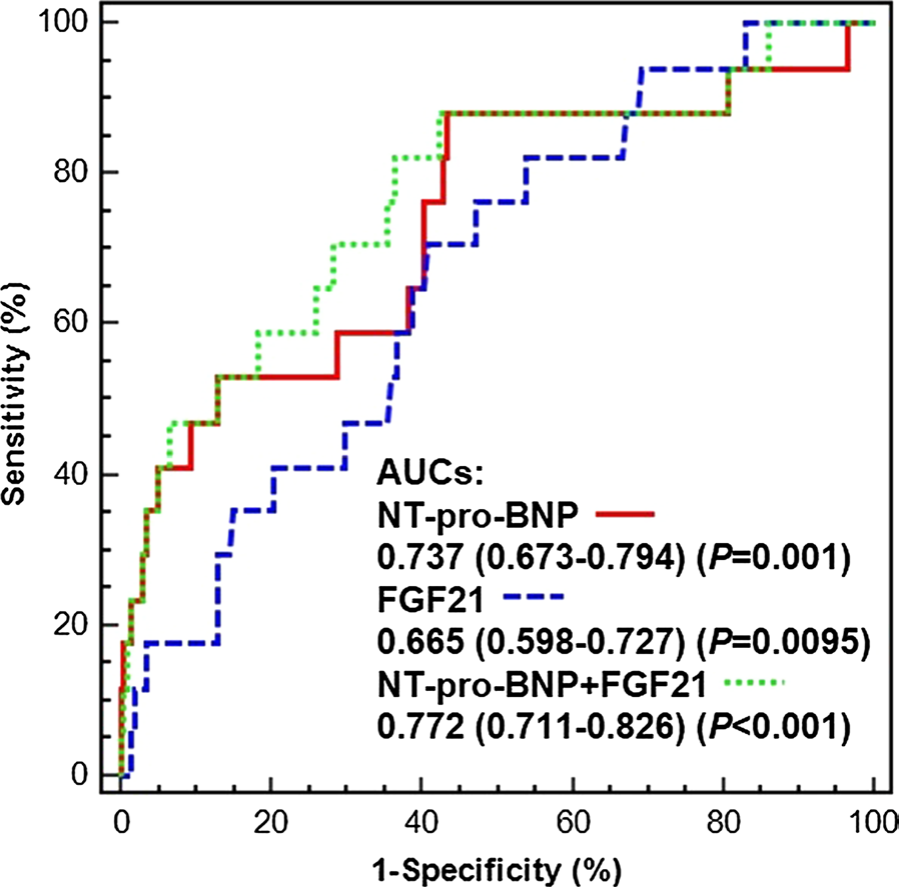
ROC analysis of the indicators used to identify left ventricular dysfunction at baseline

Receiver operating characteristic (ROC) curves were constructed to identify FGF21 and NT-pro-BNP in cases of left ventricular dysfunction at baseline. Three different ROC curves were analyzed (FGF21, NT-pro-BNP and FGF21 + NT-pro-BNP, see Fig. 1). The optimal cutoff value of FGF21 for the identification of left ventricular dysfunction at baseline was 321.5 pg/mL with a corresponding sensitivity of 70.59% and a specificity of 59.20%. The AUC was 0.665 (0.598–0.727) (*P* = 0.0095). The optimal cutoff value of NT-pro-BNP for the identification of left ventricular dysfunction at baseline was 131.3 ng/L with a corresponding sensitivity of 88.24% and a specificity of 56.72%. The AUC was 0.737 (0.673–0.794) (*P* = 0.001). The sensitivity and specificity of FGF21 combined with NT-pro-BNP for the identification of left ventricular dysfunction at baseline were 82.35% and 63.68%, respectively, with an AUC of 0.772 (0.711–0.826) (*P* < 0.001). However, pairwise comparison analysis showed that there were no significant differences among the three AUCs (FGF21 vs NT-pro-BNP, *P* = 0.4739; FGF21 vs FGF21 + NT-pro-BNP, *P* = 0.2269; NT-pro-BNP vs FGF21 + NT-pro-BNP, *P* = 0.078).

### Independent risk factors of left ventricular dysfunction at baseline

To evaluate the independent risk factors of left ventricular dysfunction at baseline, we conducted a logistic regression analysis in two models. Model 1 was a univariate analytic model including higher FGF21 (defined according to the cutoff mentioned above), higher NT-pro-BNP (defined according to the cutoff mentioned above), and gender, age, smoker, family history of CVD, diabetes, hypertension and dyslipidemia evaluated separately. The results showed that only higher FGF21 [OR 3.483 (1.182–10.262), *P* = 0.024] and higher NT-pro-BNP [OR 9.828 (2.189–44.113), *P* = 0.003] were significantly correlated with left ventricular systolic dysfunction at baseline. After multivariate analysis of both variables included in the regression (model 2), we found that a significant independent correlation between the variables and left ventricular systolic dysfunction at baseline still existed [OR 3.138 (1.037–9.500), P = 0.043; OR 9.207 (2.036–41.643), P = 0.004, as seen in Table 2].

**Table 2:** Logistic regression analysis of baseline systolic dysfunction

Variables	OR (95% CI)	P value
Model 1 (univariate)		
Higher FGF21	3.483 (1.182–10.262)	0.024
Higher NT-pro-BNP	9.828 (2.189–44.113)	0.003
Gender	1.043 (0.370–2.942)	0.936
Age	0.966 (0.919–1.015)	0.169
Smoking	1.387 (0.514–3.742)	0.518
History of CVD	0.387 (1.122–1.229)	0.107
Diabetes	0.036 (0.379–2.836)	0.944
Hypertension	1.464 (0.404–5.310)	0.562
Dyslipidemia	1.867 (0.235–14.796)	0.555
NAFLD	1.955 (0.708–5.397)	0.196
Model 2 (multivariate including both higher FGF21 and higher NT-pro-BNP)		
Higher FGF21	3.138 (1.037–9.500)	0.043
Higher NT-pro-BNP	9.207 (2.036–41.643)	0.004

Variables adjusted in model 2: gender, age, smoking, history of CVD, diabetes, hypertension, dyslipidemia, and NAFLD

*CVD* cardiovascular diseases, *FGF21* fibroblast growth factor 21, *NT*-*pro*-*BNP* N-terminal pro-brain natriuretic peptide, *NAFLD* non-alcoholic fatty liver disease

### Identification of FGF21 and NT-pro-BNP in cases of cardiac death

According to the electronic medical history system of Shanghai Jiao Tong University Affiliated Sixth People’s Hospital and telephone follow-up visits, this study recorded 9 events of cardiac death, which were all due to heart failure at a median time of follow-up year 5.

**Fig. 2:**
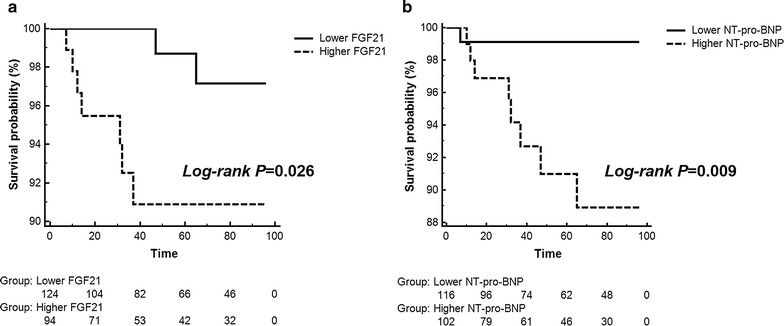
Kaplan–Meier survival analysis of FGF21 (**a**) and NT-pro-BNP (**b**) for cardiac death

We separated the patients into subgroups according to the cutoff identifying left ventricular systolic dysfunction to determine whether the same cutoff could identify cardiac death. Further Kaplan–Meier survival curves were constructed, and we found that compared with the lower FGF21 group, the higher FGF21 group suffered a greater risk of cardiac death [RR 5.000 (1.326–18.861), *P* = 0.026, as seen in Fig. 2a]. Similarly, the higher NT-pro-BNP group suffered a greater risk of cardiac death compared with the lower NT-pro-BNP group [RR 9.643 (2.596–35.825), *P* = 0.009, as seen in Fig. 2b].

Finally, we did a Cox multivariate analysis to adjust survival for confounding factors (Table 3). The results showed that after adjusting for sex, age, BMI, FPG, 2hPG, HbA1c, TG, HDL-c, LDL-c, SBP, DBP, and CRP, both FGF21 [HR 10.562 (1.388–80.404), *P* = 0.023] and NT-pro-BNP [HR 4.201 (1.470–12.008), *P* = 0.007] remained significant in identifying future risk of cardiac death.

**Table 3:** Multivariate Cox proportion hazards regression analysis for cardiac death

Baseline variables	HR (95% CI)	P value
FGF21^a^	10.562 (1.388–80.404)	0.023
NT-pro-BNP^a^	4.201 (1.470–12.008)	0.007

Confounding factors were adjusted (sex, age, BMI, FPG, 2hPG, HbA1c, TG, HDL-c, LDL-c, SBP, DBP, and CRP)

*FGF21* fibroblast growth factor 21, *NT*-*pro*-*BNP* N-terminal pro-brain natriuretic peptide

^a^Both FGF21 and NT-pro-BNP were log-transformed

## Discussion

We determined the relationship between left ventricular systolic dysfunction and FGF21, as well as the traditional indicator NT-pro-BNP, and the identification of these two variables in cases of cardiac death in both cross-sectional and follow-up analysis. We found that FGF21 and NT-pro-BNP were both correlated with left ventricular systolic dysfunction at baseline. Both higher FGF21 and higher NT-pro-BNP levels could predict cardiac death within five years. We therefore concluded that FGF21 was non-inferior to NT-pro-BNP at identifying both left ventricular systolic dysfunction at baseline and cardiac death within five years.

FGF21 is involved in the pathogenesis of cardiovascular diseases and metabolic disorders [11]. FGF21 is confirmed secreted mainly in liver and is characterized by their reduced binding affinity for heparin that enables them to be transported in the circulation to act as a hormone-like cytokine. Its action is not tissue specific but general in vivo [12]. In our previous study, patients diagnosed with coronary artery disease by coronary angiography had higher levels of FGF21. This relationship was independent of traditional risk factors [4]. As was reported in other studies, FGF21 was found to be significantly correlated with subclinical atherosclerosis and lower extremity atherosclerosis in type 2 diabetes patients [13, 14]. FGF21 was presented as a potential biomarker for cardiovascular diseases. In a clinical trial named the FIELD study [15], FGF21 was found to effectively identify the risk of cardiac events. Higher FGF21 levels at baseline could predict overall cardiac outcomes within five years. However, baseline FGF21 levels were not reported to be associated with CVD mortality in the FIELD study, probably because the subjects of that study were type 2 diabetes patients only. In addition, serum FGF21 was reported to be elevated in newly diagnosed T2DM, and positively correlated with carotid and iliac lesions in patients with subclinical atherosclerosis, especially in women [16]. Among youths at risk for metabolic syndrome, FGF21 levels were positively correlated with obesity traits, blood pressure, and lipid profiles [17], indicating its essential role in the metabolic system.

Few studies have reported the relationship between FGF21 and cardiac function as evaluated by echocardiography. Chou et al. [18] reported that FGF21 was associated with diastolic dysfunction in heart failure patients with preserved ejection fractions, including 95 patients with diastolic dysfunction and 143 controls. Multiple logistic regression analysis showed that FGF 21 was independently associated with diastolic dysfunction. Both FGF21 and NT-pro-BNP levels could indicate major adverse cardiac events after one year of follow up. In another study of 1668 patients with CAD reported by Li et al. [19], serum FGF21 levels at baseline were associated with all-cause mortality and with CVD mortality in a U-shaped pattern at a median follow-up of 4.9 years, indicating that patients with both higher and lower FGF21 levels could suffer greater risks of death. Both quartile 1 (120.8 pg/mL) and quartile 3 (357.0 pg/mL) patients had significant higher risks of CVD mortality. In our study, FGF21 was not inferior to NT-pro-BNP at identifying left ventricular systolic dysfunction at baseline. Further analysis showed that, at a median follow up of 5.0 years, patients with higher FGF21 levels (≥321.5 pg/mL) at baseline are at higher risk of CVD mortality, which was also not inferior to NT-pro-BNP. The corresponding cutoff of FGF21 in our study was similar to that of Li Q, et al. Our findings are also consistent with the results of Chou et al. and are more typical of the association between FGF21 and LVEF, as Chou et al. excluded patients with LVEF <45%.

Ventricular remodeling is an adaptive response caused by the hypertrophy of myocardial cells during stress. Mature myocardial cells are differentiated cells that cannot regenerate. Myocardial cells tend to be hypertrophic rather than differentiated during stress, resulting from increased amounts of extracellular matrix and fibroblasts in the heart [12]. FGF21, as a novel cytokine, is involved in this pathogenesis. In rodent models, the expression of FGF21 in cardiac cells can be up-regulated by the hypertrophy of myocardial cells, which is also regulated by the SIRT1/PPARα pathway in the liver [20]. The mass of the heart tends to be larger, with deteriorating cardiac function in FGF21 knock-out rats after continuous infusion of isoprenaline [21]. Moreover, FGF21 were also shown to play an important role in oxidative stress [22]; FGF21 deletion aggravates aortic remodeling and cell death probably via exacerbation of aortic inflammation and oxidative stress [23]. In our study, FGF21 can identify left ventricular systolic dysfunction, which may be due to the above-mentioned mechanism. The secretion of FGF21 via a compensatory response may contribute to these results.

There are some limitations to this study. First, the sample size of this study is small and we did not calculate the sample size at baseline. Our preliminary results should be validated in further studies with a larger sample size, which can provide sufficient statistical power. Second, the subjects enrolled in our study at baseline are all from the department of cardiology, and tend to be elderly. We also intend to recruit subjects from different age ranges to generalize our results. Finally, no relationship between FGF21 and age is observed at baseline, which is not consistent with another study [15]. Patients do not undergo additional FGF21 measurements during their follow up so that we cannot further clarify the exact relationship between FGF21 and age.

In conclusion, we may consider serum FGF21 levels to be correlated with left ventricular systolic dysfunction at baseline. Patients with higher FGF21 levels at baseline are at greater risk of CVD mortality. The role of FGF21 in both left ventricular systolic dysfunction and cardiac death is not inferior to that of NT-pro-BNP.

## Abbreviations

BMI: body mass index
BP: blood pressure
CAD: coronary artery disease
CI: confidence interval
CRP: C-reactive protein
FGF: fibroblast growth factor
FPG: fasting plasma glucose
FS: shortening fraction
HbA1c: hemoglobin A1c
HDL-c: high-density lipoprotein cholesterol
HOMA-IR: homeostasis model assessment-insulin resistance
HR: hazard ratio
ISH: international society of hypertension
IVST: interventricular septum thickness
LA: left atrium
LDL-c: low-density lipoprotein cholesterol
LV: left ventricular
LVEF: left ventricular ejection fraction
LVSd: left ventricular end-systolic diameter
LVDd: left ventricular end-diastolic diameter
NAFLD: non-alcoholic fatty liver disease
NT-pro-BNP: N-terminal pro-brain natriuretic peptide
PWT: posterior wall thickness
ROC: receiver operating characteristic
TC: total cholesterol
TG: triglyceride
WHO: World Health Organization
2-h PG: 2-h postprandial plasma glucose
